# miR-140-3p suppresses the proliferation and migration of macrophages

**DOI:** 10.1590/1678-4685-GMB-2021-0160

**Published:** 2022-06-15

**Authors:** Pingping Qiao, Jun Zhu, Xiaoheng Lu, Yifei Jin, Yifan Wang, Qianqian Shan, Yaxian Wang

**Affiliations:** 1 Nantong University, Key Laboratory of Neuroregeneration of Jiangsu and Ministry of Education, Co-innovation Center of Neuroregeneration, NMPA Key Laboratory for Research and Evaluation of Tissue Engineering Technology Products, Nantong, Jiangsu, China.; 2 The Affiliated Hospital of Nantong University, Department of Thoracic Surgery, Nantong, Jiangsu, China.; 3 Medical School of Nantong University, Nantong, Jiangsu, China.; 4 The Affiliated Hospital of Nantong University, Department of Radiotherapy and Oncology, Nantong, Jiangsu, China.

**Keywords:** Peripheral nerve injury, miR-140-3p, macrophage, proliferation, migration

## Abstract

Macrophages benefit myelin debris removal, blood vessel formation, and Schwann cell activation following peripheral nerve injury. Identifying factors that modulate macrophage phenotype may advantage the repair and regeneration of injured peripheral nerves. microRNAs (miRNAs) are important regulators of many physiological and pathological processes, including peripheral nerve regeneration. Herein, we investigated the regulatory roles of miR-140-3p, a miRNA that was differentially expressed in injured rat sciatic nerves, in macrophage RAW264.7 cells. Observations from EdU proliferation assay demonstrated that elevated miR-140-3p decreased the proliferation rates of RAW264.7 cells while suppressed miR-140-3p increased the proliferation rates of RAW264.7 cells. Transwell-based migration assay showed that up-regulated and down-regulated miR-140-3p led to elevated and reduced migration abilities, respectively. However, the abundances of numerous phenotypic markers of M1 and M2 macrophages were not significantly altered by miR-140-3p mimic or inhibitor transfection. Bioinformatic analysis and miR-140-3p-induced gene suppression examination suggested that *Smad3* might be the target gene of miR-140-3p. These findings illuminate the inhibitory effects of miR-140-3p on the proliferation and migration of macrophages and contribute to the cognition of the essential roles of miRNAs during peripheral nerve regeneration.

## Introduction

Peripheral nerves, unlike central nerves, exhibit a remarkable spontaneous regeneration capacity after nerve injury. The successful regeneration of injured peripheral nerves largely depends on the reprogramming of Schwann cells, the attraction and recruitment of macrophages, and the activation of the intrinsic growth capacity of affected neurons (Chen Z-L *et al.,* 2007; Jessen and Mirsky, 2016). Following peripheral nerve injury, infiltrated macrophages clear redundant myelin debris and eliminate inhibiting factors for subsequent axon growth (Chen P *et al.,* 2015). Besides their phagocytosis function, macrophages sense hypoxia, encourage angiogenesis, and promote the proliferation and directional migration of Schwann cells (Cattin *et al.,* 2015; Liu *et al.,* 2019). Therefore, regulating the biological activities of macrophages may facilitate the regeneration of injured peripheral nerves.

Emerging studies have demonstrated that non-coding RNAs, especially microRNAs (miRNAs), play essential roles following peripheral nerve injury (Yu *et al.,* 2015). miRNAs are evolutionarily conserved single-stranded non-coding RNAs containing ~22 nucleotides (Bartel, 2018). miRNAs pair with complementary sequences within their target mRNAs and thus silence their target mRNAs post-transcriptionally (Bartel, 2009). A large number of miRNAs have been found to be dysregulated in peripheral nerves after nerve injury, implying the involvement of miRNAs in the peripheral nerve repair and regeneration process (Li *et al.,* 2011; Yu *et al.,* 2011). Further functional studies have demonstrated that many differentially expressed miRNAs participate in the modulation of various types of cells in peripheral nerves, such as Schwann cells, neurons, and endothelial cells (Yi *et al.,* 2016; 2017; Ji *et al.,* 2019; Wang X *et al.,* 2019). Therefore, it is likely that differentially expressed miRNAs following peripheral nerve injury may also regulate the cellular behaviors of macrophages.

Sequencing data of injured rat sciatic nerves showed that miR-140-3p, a miRNA previously annotated as miR-140* according to miRbase database (Ambros *et al.,* 2003), was one of the most highly expressed and significantly dysregulated miRNAs in the injured peripheral nerves (Yu *et al.,* 2011). However, whether differentially expressed miR-140-3p modulates the phenotype of macrophages remains largely unrevealed. Therefore, in the current study, we cultured macrophage RAW264.7 cells, transfected macrophages with miR-140-3p mimic or inhibitor to increase or decrease miR-140-3p abundance, respectively, and examined the functional roles of miR-140-3p in the proliferation, migration, and polarization of macrophages. Potential targets of miR-140-3p were also investigated using bioinformatic tools and expression correlation analysis.

## Material and Methods

### Cell culture and transfection

The murine macrophage cell line RAW264.7 was purchased from the Typical Culture Preservation Commission (Chinese Academy of Sciences, Shanghai Institutes for Biological Sciences Cell Resource Center, Shanghai, China). RAW264.7 cells were cultured in DMEM (Corning, Coring, NY, USA) supplemented with 10% heat-inactivated FBS (Gibco, Grand Island, NY, USA) in a 5% CO_2_ incubator at 37°C. Cultured RAW264.7 cells were transfected with miR-140-3p mimic, miR-140-3p inhibitor, or corresponding non-targeting controls (RiboBio, Guangzhou, Guangdong, China) using Lipofectamine RNAiMAX transfection reagent (Invitrogen, Carlsbad, CA, USA).

### Quantificational real time PCR (qRT-PCR)

RNA samples were isolated from cultured RAW264.7 cells using RNA-Quick Purification Kit (Yishan Biotechnology Co., LTD, Shanghai, China) and reversely transcribed using Bulge-Loop^TM^ miRNA qRT-PCR Starter Kit (RiboBio) or HiScript III RT SuperMix for qPCR (+gDNA wiper) (Vazyme, Nanjing, Jiangsu, China). A StepOne Real-time PCR System (Applied Biosystems, Foster City, CA, USA) was used to determine the Ct values of miR-140-3p, miRNA internal control *U6*, *Cd86*, *Tnf*, *Gpr18*, *Egr2*, *Myc*, *Il-10*, *Smad3*, and mRNA internal control *Gapdh*. The comparative 2^-ΔΔCt^ method was applied to calculate relative gene expressions. Bulge-loop^TM^ miRNA qRT-PCR Primer Sets specific for miR-140-3p was designed and synthesized by RiboBio and primer sets for coding RNAs were synthesized by Sangon Biotech (Shanghai, China). Primer sequences (Table 1) were designed using Primer Express® software (v3.0.1; Thermo Fisher Scientific, Inc.). The amplification efficiency of primer pairs was determined by producing serial dilutions of the DNA sample. Primers with amplification efficiencies between 90% and 110% were selected (Seol *et al.,* 2011).

**Table 1.** Nucleotide sequences of the primers used in qRT-PCR.

Gene Symbol	Accession Number	Primer sequences (5'-3')	Length (bp)	Efficiency (%)
*Cd86*	XM_011245812.3	Sense: GGCTTGGCAATCCTTATCTTT Antisense: ACATCTTCTTAGGTTTCGGGTG	487	95
*Tnf*	NM_001278601.1	Sense: GTCAGGTTGCCTCTGTCTCA Antisense: TCAGGGAAGAGTCTGGAAAG	82	94
*Gpr18*	NM_182806.2	Sense: CACCCTGAGCAATCACAACC Antisense: CAGCACTAATGAAAGCAAGAAGC	347	108
*Egr2*	XM_030244870.2	Sense: ACCTCCTTCCTACCCATCCC Antisense: CAGAGCGTGAGAACCTCCTATC	443	107
*Myc*	NM_001177353.1	Sense: AGGACTGTATGTGGAGCGGTTTC Antisense: TGCTGTCGTTGAGCGGGTAG	216	99
*Il-10*	NM_010548.2	Sense: CTTTGCTATGGTGTCCTTTCA Antisense: AAGACCCATGAGTTTCTTCAC	81	98
*Smad3*	XM_006510821.5	Sense: CGTGGAACTTACAAGGCGACA Antisense: GGGAGACTGGACGAAAATAGC	109	94
*Gapdh*	XM_036165840.1	Sense: CCTTCATTGACCTCAACTACATG Antisense: CTTCTCCATGGTGGTGAAGAC	215	99

### Cell viability assay

Cell counting kit-8 (CCK-8) proliferation assay kit (Beyotime Biotechnology, Shanghai, China) was used to determine the effect of miR-140-3p on the viability of cells according to the manufacturer’s instructions. Briefly, RAW264.7 cells were transfected with miR-140-3p mimic, mimic control, miR-140-3p inhibitor, or inhibitor control for 36 hours, respectively. Then cells were seeded onto a 96-well cell culture plate and treated with 10 μl CCK-8 solution for 2 hours (Shen *et al.,* 2020). A microplate reader (Bio-Rad Laboratories, Inc., Hercules, CA, USA) was applied to measure the optical density (O.D.) readings at 450 nm and to calculate the relative number of viable RAW264.7 cells in the medium.

### Cell proliferation assay

Cell-Light^TM^ EdU DNA Cell Proliferation Kit (RiboBio) was applied to examine the effect of miR-140-3p on the proliferation rate of cells as previously reported (Bei *et al.,* 2016). Briefly, after transfection with miR-140-3p mimic, miR-140-3p inhibitor, or corresponding non-targeting controls for 36 hours, RAW264.7 cells were exposed to 50 μM EdU (RiboBio) for 2 hours and fixed with 4% formaldehyde (Xilong Scientific, Guangzhou, China) in PBS for 30 minutes. Cells were labeled with Hoechst 33342 staining in blue color while proliferative cells were labeled with EdU staining in red color. Images were taken under a DMR fluorescence microscope (Leica Microsystems, Bensheim, Germany) and the relative proliferation rate of RAW264.7 cells was calculated by dividing the number of proliferative RAW264.7 cells to the number of total cells in randomly selected fields.

### Cell migration assay

A transwell-based cell migration assay was conducted to evaluate the effect of miR-140-3p on the migration ability of RAW264.7 cells. RAW264.7 cells transfected with miR-140-3p mimic, miR-140-3p inhibitor, or corresponding non-targeting controls were resuspended in DMEM. Cell suspension was added to the upper chamber of a 6.5 mm transwell chamber with 8 μm pores (Costar, Cambridge, MA, USA) while FBS containing complete cell culture medium was added to the lower chamber. After 36 hours of culture, RAW264.7 cells left on upper surface of the upper chamber were scraped away with a cotton swab while invaded cells on the bottom surface were fixed in 4% paraformaldehyde, stained with 0.1% crystal violet, and observed under a DMR inverted microscope (Leica Microsystems). The relative migration ability of RAW264.7 cells was calculated by measuring crystal violet-stained areas in randomly selected fields. In addition, cell migration was confirmed with a second assay using fibronectin-coated transwells. The procedure was the same as above except that the surface of the transwell chamber was pre-coated with a layer fibronectin (EMD Millipore Corporation, Temecula, CA, USA) before cells plantation, as previously reported (Cougoule *et al.,* 2010; Börschel *et al.,* 2020).

### Bioinformatic analysis of potential target genes of miR-140

The prediction of potential target genes of mmu-miR-140 and the involvement of Gene Ontology (GO) terms and Kyoto Enrichment of Genes and Genomes (KEGG) pathways were analyzed using the ClueGo plug-in in the Cytoscape software (Shannon *et al.,* 2003). Enriched GO terms and KEGG pathways of potential target genes *Smad3*, *Cxcl12*, and *Hdac4* with a p-value cutoff of 0.05 were selected. An interaction network of mmu-miR-140, potential target genes *Smad3*, *Cxcl12*, and *Hdac4*, and potential target gene-involved GO terms and KEGG pathways were constructed using the Cytoscape software.

### Statistical analysis

Statistical analysis was conducted by the Student's *t*-test or one-way analysis of variance (ANOVA) test using GraphPad Prism 5.0 (GraphPad Software, Inc., San Diego, CA, USA). Differences with a p-value less than 0.05 were considered statistically significant.

## Results

### miR-140-3p affects cell viability of macrophages

**Figure 1 -:**
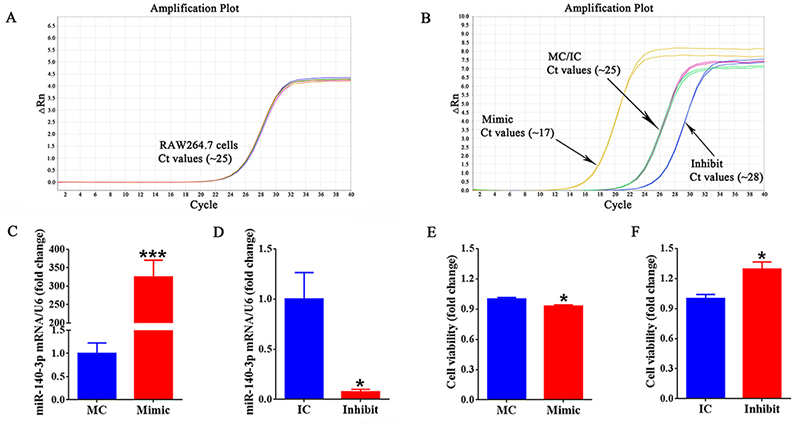
Effect of miR-140-3p on macrophage viability. **(A)** The amplification plot of miR-140-3p in RAW264.7 cells. **(B)** The amplification plot of miR-140-3p in RAW264.7 cells transfected with miR-140-3p mimic (Mimic), mimic control (MC), inhibitor (Inhibit), or inhibitor control (IC), respectively. **(C)** The relative abundances of miR-140-3p in RAW264.7 cells after transfection with mimic control (MC) or miR-140-3p mimic (Mimic). **(D)** The relative abundances of miR-140-3p in RAW264.7 cells after transfection with inhibitor control (IC) or miR-140-3p inhibitor (Inhibit). **(E)** The cell viability of RAW264.7 cells transfected with mimic control (MC) or miR-140-3p mimic (Mimic). **(F)** The cell viability of RAW264.7 cells transfected with inhibitor control (IC) or miR-140-3p inhibitor (Inhibit). All data were summarized from 3 paired experiments (n=3). Numerical data were presented as means ± SEM. Asterisk represents statistically different from control (*** indicates p-value < 0.001, * indicates p-value < 0.05).

Cultured macrophage RAW264.7 cells were transfected with miR-140-3p mimic, mimic control, miR-140-3p inhibitor, or inhibitor control to determine the functional effects of miR-140-3p on macrophages. Compared with RAW264.7 cells or cells transfected with mimic control, RAW264.7 cells transfected with miR-140-3p mimic exhibited robust elevation of miR-140-3p expression (Figure 1A-C). In contrast, RAW264.7 cells transfected with miR-140-3p inhibitor showed a vigorous reduction of the abundance of miR-140-3p (Figure 1A, B, D), indicating the high efficiency of cellular transfection. We then examined the effect of miR-140-3p on macrophage viability. The results showed that compared with mimic control, miR-140-3p mimic significantly decreased the cell viability (Figure 1E). While miR-140-3p inhibitor significantly increased cell viability compared with the control group (Figure 1F).

### miR-140-3p inhibits the proliferation of macrophages

**Figure 2-:**
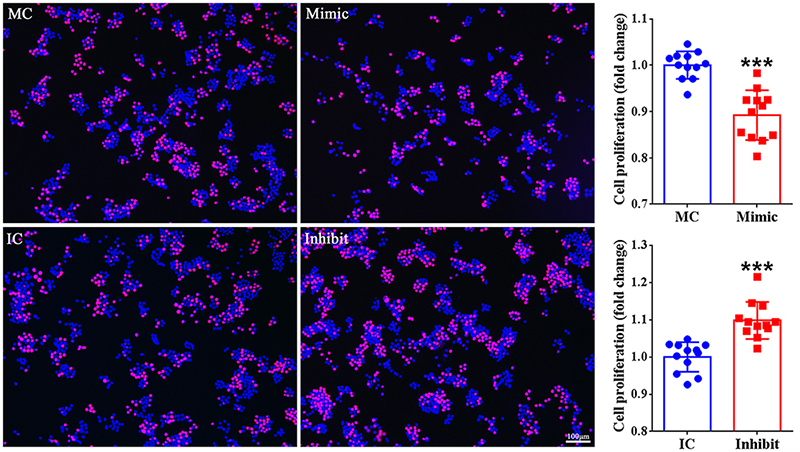
Effect of miR-140-3p on macrophage proliferation. Representative EdU staining images of RAW264.7 cells transfected with mimic control (MC), miR-140-3p mimic (Mimic), inhibitor control (IC), or miR-140-3p inhibitor (Inhibit). The proliferation rates of RAW264.7 cells were determined as the relative ratio of EdU-positive cells to total cells in the experiment group as compared with the corresponding control group and the cell proliferation assay was repeated four times using triplicate wells (n=12). Numerical data were presented as means ± SD. Red color represents EdU staining and blue color represents Hoechst 33342 staining. Scale bars represents 100 μm. Asterisk represents statistically different from control (*** indicates p-value < 0.001).

The effect of miR-140-3p on macrophage proliferation was then investigated. Considerable cells were observed to be existed in a proliferating state in RAW264.7 cells transfected with mimic control or inhibitor control. Compared with cells transfected with mimic control, less amount of RAW264.7 cells were labeled as EdU-positive cells. Rather, the number of EdU-positive RAW264.7 cells was obviously higher in cells transfected with miR-140-3p inhibitor as compared with cells transfected with inhibitor control (Figure 2). The inhibiting role of miR-140-3p mimic and the promoting role of miR-140-3p inhibitor in RAW264.7 cell proliferation suggest that miR-140-3p suppresses the proliferation of macrophages.

### miR-140-3p inhibits the migration of macrophages

**Figure 3-:**
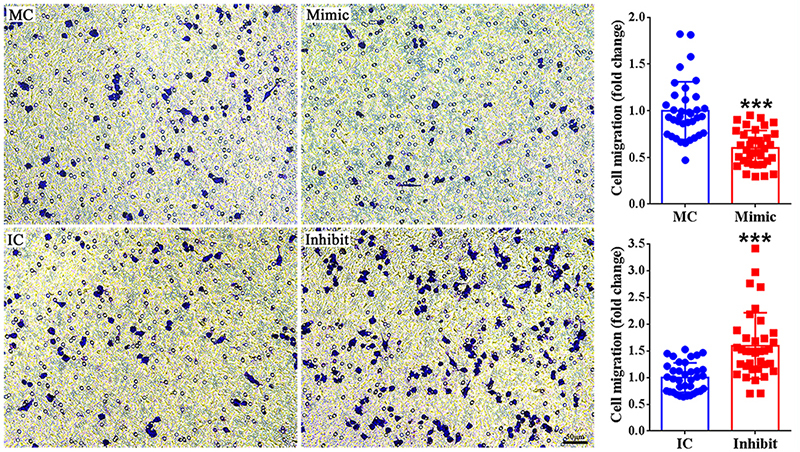
Effect of miR-140-3p on macrophage migration. Representative transwell migration images of RAW264.7 cells transfected with mimic control (MC), miR-140-3p mimic (Mimic), inhibitor control (IC), or miR-140-3p inhibitor (Inhibit). The migration abilities of RAW264.7 cells were determined as the relative crystal violet-stained areas in the experiment group as compared with the corresponding control group. All data were summarized from 3 paired experiments (n=3). Numerical data were presented as means ± SD. Scale bars represents 50 μm. Asterisks represent statistical differences from control (*** indicates p-value < 0.001).

**Figure 4 -:**
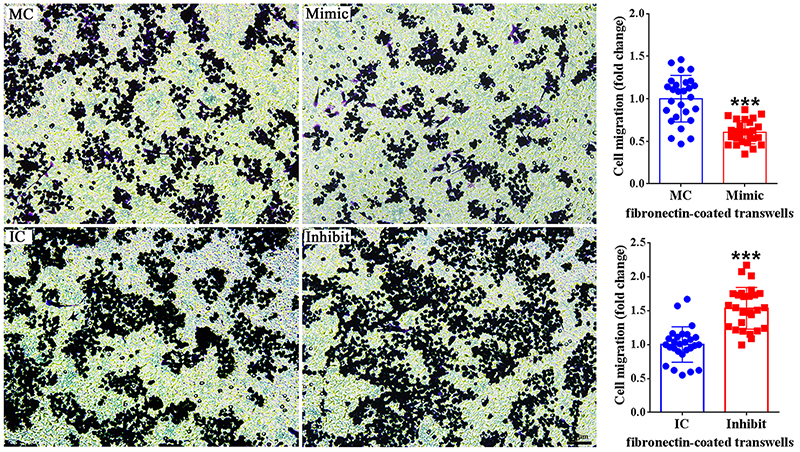
Effect of miR-140-3p on macrophage migration through fibronectin-coated transwells. Representative transwell migration images of RAW264.7 cells transfected with mimic control (MC), miR-140-3p mimic (Mimic), inhibitor control (IC), or miR-140-3p inhibitor (Inhibit). All data were summarized from 3 paired experiments (n=3). Numerical data were presented as means ± SD. Scale bars represents 50 μm. Asterisks represent statistical differences from control (*** indicates p-value < 0.001).

Transwell migration assay was applied to observe the effect of miR-140-3p on macrophage migration. Crystal violet staining of the bottom surface of the upper chamber of transwell showed that RAW264.7 cells obtain certain ability to migrate across transwell. However, following the transfection of miR-140-3p mimic, crystal violet-stained areas of migrated cells reduced to about 60% as compared with stained areas in the control group. In contrast, RAW264.7 cells transfected with miR-140-3p inhibitor had enlarged crystal violet-stained areas (Figure 3). Additionally, we obtained similar results in the second fibronectin-coated transwells assay (Figure 4). These observations indicate that besides the proliferation of macrophages, miR-140-3p also exhibit inhibitory effect on the migration of macrophages.

### miR-140-3p mimic reverses the effect of the inhibitor

**Figure 5-:**
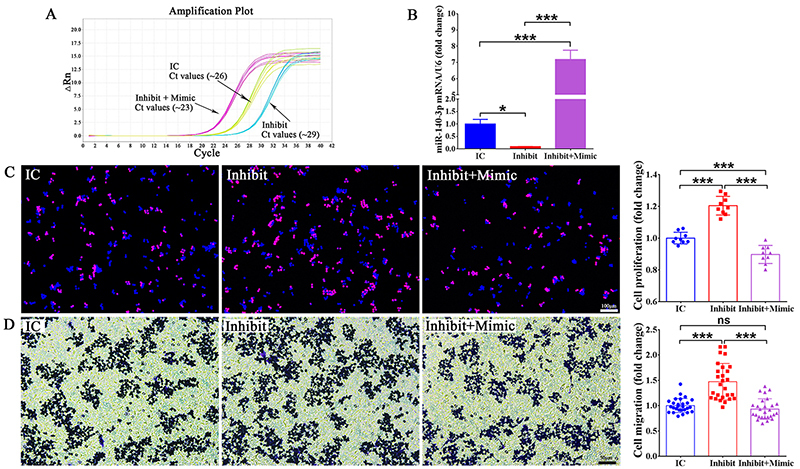
miR-140-3p mimic reverses the effect of the inhibitor. **(A)** The amplification plot of miR-140-3p in RAW264.7 cells transfected with miR-140-3p inhibitor (Inhibit), inhibitor control (IC) or miR-140-3p inhibitor+mimic (Inhibit+Mimic), respectively. **(B)** The relative abundances of miR-140-3p in RAW264.7 cells after transfection with inhibitor control (IC), miR-140-3p inhibitor (Inhibit) or miR-140-3p inhibitor+mimic (Inhibit+Mimic). **(C)** Representative EdU staining images of RAW264.7 cells transfected with inhibitor control (IC), miR-140-3p inhibitor (Inhibit) or miR-140-3p inhibitor+mimic (Inhibit+Mimic), Scale bars represents 100 μm. **(D)** Representative transwell migration images of RAW264.7 cells transfected with inhibitor control (IC), miR-140-3p inhibitor (Inhibit) or miR-140-3p inhibitor+mimic (Inhibit+Mimic), Scale bars represents 50 μm. All data were summarized from 3 paired experiments (n=3). Numerical data were presented as means ± SD. Asterisks represent statistical differences between groups (*** indicates p-value < 0.001, * indicates p-value < 0.05).

Quantificational results showed that miR-140-3p mimic significantly increased the expression level of miR-140-3p (over 300 fold). So, we wondered if miR-140-3p mimic could reverse the effect of the inhibitor when RAW264.7 cells were transfected with both. PCR results showed that miR-140-3p inhibitor significantly reduced the expression level of miR-140-3p which was consistent with the above results. However, miR-140-3p mimic reversed the effect of inhibitor and elevated the abundance of miR-140-3p when RAW264.7 cells were simultaneously transfected with miR-140-3p inhibitor and mimic (Figure 5A, B). Cellular behavior evaluation showed that miR-140-3p mimic reversed the ability of miR-140-3p inhibitor to promote cell proliferation and migration (Figure 5C, D).

### miR-140-3p does not affect the polarization of macrophages

**Figure 6-:**
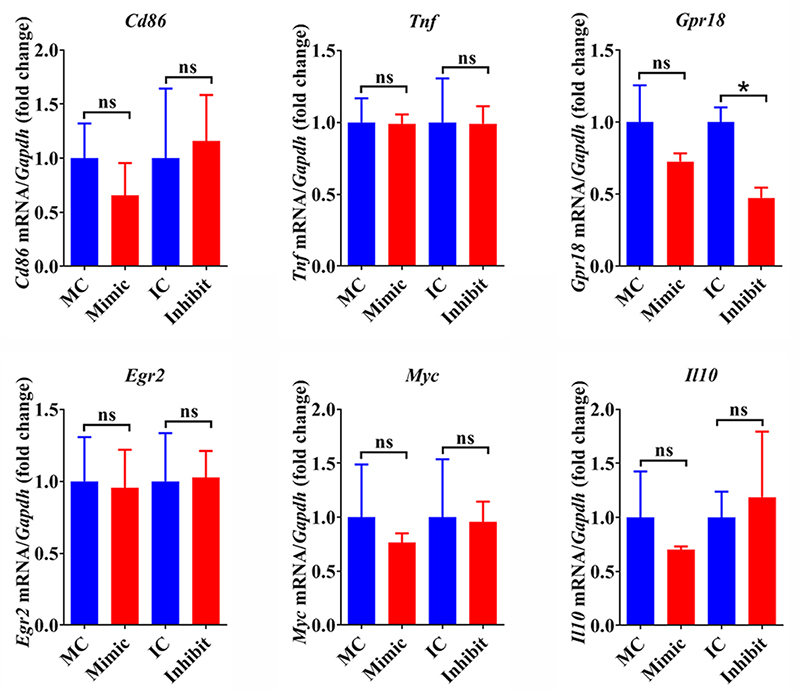
Effect of miR-140-3p on macrophage polarization. The relative abundances of *Cd86*, *Tnf*, *Gpr18*, *Egr2*, *Myc*, and *Il10* in RAW264.7 cells after transfection with mimic control (MC), miR-140-3p mimic (Mimic), inhibitor control (IC), or miR-140-3p inhibitor (Inhibit). Gene abundances were determined as the relative expressions in the experiment group as compared with the corresponding control group and summarized from 3 paired experiments (n=3). Numerical data were presented as means ± SEM. Asterisks represent statistical differences from control (* indicates p-value < 0.05) and ns represents non-statistically relevant to the control group.

Pro-inflammatory (M1) and anti-inflammatory (M2) macrophages execute unique functions in diverse biological activities. The switch of M1 and M2 phenotypes of macrophages is also involved in the regeneration of injured peripheral nerves. Therefore, the expression levels of M1 and M2 macrophage markers in miR-140-3p-transfected RAW264.7 cells were evaluated to identify whether miR-140-3p would affect the M1/M2 polarization of macrophages. RT-PCR results showed that neither miR-140-3p mimic nor miR-140-3p inhibitor significantly altered the abundances of mRNAs coding for M1/M2 markers *Cd86* and *Tnf* or M2 macrophage markers *Egr2*, *Myc*, and *Il10*. For M1 macrophage marker *Gpr18*, although the mRNA expression of *Gpr18* seemed to be decreased after miR-140-3p inhibitor transfection, the expression of *Gpr18* was not increased after miR-140-3p mimic transfection (Figure 6). These outcomes imply that miR-140-3p may not induce changes of M1 and M2 phenotypes of macrophages.

### Identification of potential targets of miR-140

**Figure 7 -:**
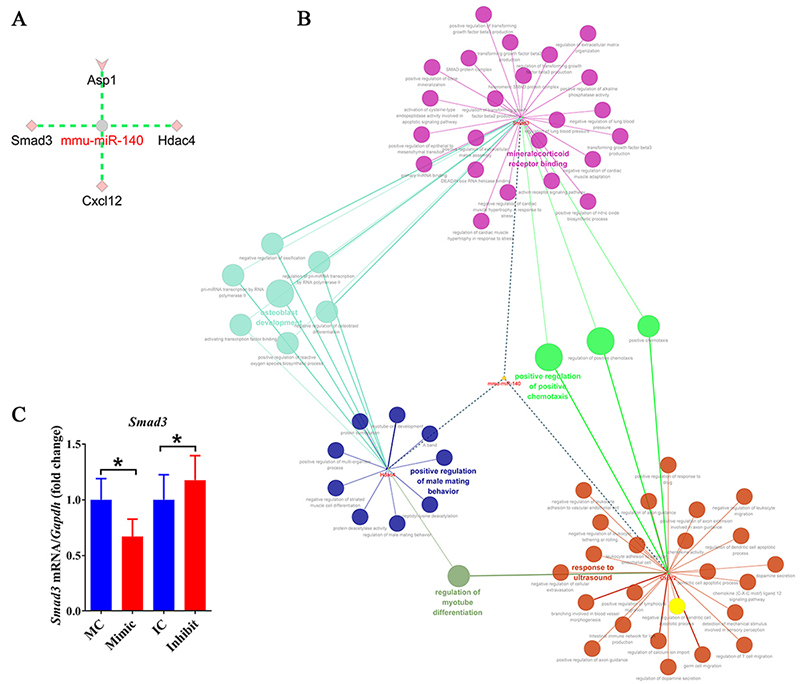
Candidate target genes of miR-140. **(A)** Predicted potential target genes of mmu-miR-140. **(B)** The miRNA-potential target gene-biological activity network of mmu-miR-140. Dotted lines represent interactions between mmu-miR-140 and *Smad3*, *Cxcl12*, and *Hdac4* while solid lines represent interactions between *Smad3*, *Cxcl12*, and *Hdac4* and GO terms or KEGG pathways. Diverse groups of biological functions are indicated by different colors. Node sizes reflect the enrichment of biological functions. **(C)** The relative abundances of *Smad3* in RAW264.7 cells after transfection with mimic control (MC), miR-140-3p mimic (Mimic), inhibitor control (IC), or miR-140-3p inhibitor (Inhibit). The abundances of *Smad3* were determined as the relative expressions in the experiment group as compared with the corresponding control group and summarized from 3 paired experiments (n=3). Numerical data were presented as means ± SEM. Asterisks represent statistical differences from control (* indicates p-value < 0.05).

Potential target genes of miR-140 in macrophage RAW264.7 cells were discovered using Cytoscape bioinformatic analysis software in view of the fact that miRNAs regulate biological activities via binding to and suppressing their target genes. A total of four coding genes, i.e., *Asp1*, *Smad3*, *Cxcl12*, and *Hdac4*, were identified to be potential target genes of mmu-miR-140 (Figure 7A). Downstream GO terms and KEGG pathways of these candidate target genes were identified and an interaction network between mmu-miR-140, candidate target genes of mmu-miR-140, as well as involved GO terms and KEGG pathways was generated (Figure 7B). *Asp1* was not found to be significantly related with GO terms or KEGG pathways and thus was not displayed in the interaction network. The constructed network showed that *Smad3* was associated with activities enriched in functional groups of mineralcorticoid receptor binding, osteoblasts development, and positive regulation of positive chemotaxis, *Hdac4* was associated with osteoblasts development, positive regulation of positive chemotaxis, and regulation of myotube differentiation, while *Cxcl12* was associated with regulation of myotube differentiation, response to ultrasound, and positive regulation of positive chemotaxis. Considering that *Smad3* was linked to a large number of essential biological processes, the regulating effect of miR-140-3p on *Smad3* mRNA expression was further examined. RT-PCR results demonstrated that the mRNA expression of *Smad3* was reduced after miR-140-3p mimic transfection but was increased after miR-140-3p inhibitor transfection, suggesting that miR-140-3p negatively regulates the abundance of *Smad3* mRNA (Figure 7C).

## Discussion

Functional recovery of injured peripheral nerves requires the joint work of neurons, Schwann cells, endothelial cells, macrophages, fibroblasts, and other types of cells. miRNAs extensively regulate the biological functions of various types of cells and execute considerable functions during peripheral nerve regeneration. By performing deep sequencing, the expression patterns of miRNAs in the proximal nerve stumps after rat sciatic nerve injury were explored and many dysregulated miRNAs were screened, including miR-140-3p (Yu *et al.,* 2011).

Although the regulatory roles of miR-140-3p in Schwann cells have not been reported, a previous study examined the effect of miR-140-5p, a miRNA that originates from the opposite arm of the pre-miR-140, on Schwann cells and showed that miR-140-5p inhibits the differentiation and myelination of Schwann cells (Viader *et al.,* 2011). The biological functions of miR-140 on endothelial cells have also been discovered. For example, Sun *et al.* (2016) found that miR-140-5p inhibits the viability, migration, and tube formation of human umbilical vein endothelial cells Zhang H *et al.* (2020) demonstrated that miR-140-3p impairs the viability and colony formation ability of human coronary endothelial cells. Liang *et al.* (2020) demonstrated that miR-140-3p impairs the proliferation, wound healing, and tube formation of human umbilical vein endothelial cells. Here, we examined the regulatory effects of miR-140 on macrophages and found that miR-140-3p decreases the cell viability, and inhibits the proliferation and migration of macrophages. Therefore, it is possible that elevated miR-140-3p after peripheral nerve injury may play inhibitory roles in the cellular behaviors of Schwann cells, endothelial cells, and macrophages and impair the nerve regeneration process. Therefore, therapies targeting at suppressing the expression of miR-140-3p may benefit peripheral nerve regeneration.

Besides cellular proliferation and migration, the polarization of macrophages and changes of M1 and M2 macrophages are highly associated with the regeneration process (Liu *et al.,* 2019; Zhang F *et al.,* 2020). At present, a number of miRNAs have been confirmed to be involved in the regulation of macrophage polarization. For example, miR-9, miR-127, miR-155, and miR-125b have been shown to promote M1 polarization while miR-124, miR-223, miR-34a, let-7c, miR-132, miR-146a, and miR-125a-5p induce M2 polarization in macrophages (Essandoh *et al.,* 2016). To evaluate the potential effect of miR-140-3p on the polarization of M1/M2 macrophages, the mRNA expression patterns of canonical macrophage markers as well as novel murine M1 macrophage marker *Gpr18* and novel murine M2 macrophage markers *Egr2* and *Myc* were determined (Jablonski *et al.,* 2015). Quantificational outcomes did not reveal significant alternations of the abundances of universal macrophage markers and M1/M2-exclusive macrophage markers, suggesting that miR-140-3p may fail to induce M1/M2 phenotype switch in macrophages.

The potential downstream target genes and biological implications of miR-140-3p were further analyzed. Notably, *Smad3*, a well-known intracellular signal transducer of TGF-β signaling pathway, was identified as a candidate target gene of miR-140-3p. Mojsilovic *et al*. (2018) reported that estramustine phosphate (EP) inhibited RAW264.7 cell migration by decreasing the ability of *TGF-β* to trigger the activation of its downstream *Smad3* effector. Therefore, *Smad3* is an important factor affecting cell migration. Cytoscape analyses-identified *Smad3*-associated GO terms and KEGG pathways are also closely connected with tissue development and regeneration. For instance, mineralcorticoid receptor activation adjust the pro-inflammatory signaling and regulate the injury response of cardiac tissue (Ong *et al.,* 2020). Macrophage chemotaxis, i.e., external stimulus-induced movement of macrophages, is important for the initiation of the repair and regeneration of injured tissues, including the nervous system (Calcutt *et al.,* 1994; Bosurgi *et al.,* 2017; Yano *et al.,* 2018; Wang P *et al.,* 2019). These findings, together with the inhibitory effect of miR-140-3p on *Smad3* mRNA expression, indicate that miR-140-3p may participate in macrophage phenotype modulation and peripheral nerve regeneration via negatively regulating *Smad3*, which remains to be confirmed by our further study.

Collectively, our results reveal functional effects of miR-140-3p on macrophages and suggest that overexpression and silencing of miR-140-3p inhibits and promotes the proliferation rate and migration ability of macrophages, respectively. These findings contribute to a better understanding of the essential regulatory roles of miR-140-3p in multiple cell types and emphasize the functional involvement of miRNAs in peripheral nerve repair and regeneration.
